# Plagiarism: Why is it such a big issue for medical writers?

**DOI:** 10.4103/2229-3485.80370

**Published:** 2011

**Authors:** Natasha Das, Monica Panjabi

**Affiliations:** *Freelance Medical Writer, Delhi, India*

**Keywords:** Action against plagiarism, duplicate publication, medical writing, plagiarism, unethical publication practice

## Abstract

Plagiarism is the wrongful presentation of somebody else‘s work or idea as one’s own without adequately attributing it to the source. Most authors know that plagiarism is an unethical publication practice. Yet, it is a serious problem in the medical writing arena. Plagiarism is perhaps the commonest ethical issue plaguing medical writing. In this article, we highlight the different types of plagiarism and address the issues of plagiarism of text, plagiarism of ideas, mosaic plagiarism, self-plagiarism, and duplicate publication. An act of plagiarism can have several repercussions for the author, the journal in question and the publication house as a whole. Sometimes, strict disciplinary action is also taken against the plagiarist. The article cites examples of retraction of articles, suspension of authors, apology letters from journal editors, and other such actions against plagiarism.

## INTRODUCTION

The essence of good medical writing, or for that matter, any kind of scientific writing, is a clear, concise, accurate, and honest presentation of the scientific idea. Medical writers often have to write within tight deadlines. Additionally, medico-marketing writing also involves a lot of competitive pressure to be able to present a drug, device, or healthcare service in a fair fashion.

Some of these fallacies are unintentional and easy to remedy. For example, when an article does not score high on clarity or lacks conciseness, the deficiency is typically unintentional. A writer with a good grasp of the concept and language can often amend these fallacies with some practice. This is not enough. Accuracy and honesty are very important requisites when it comes to good scientific writing.

## PLAGIARISM

Perhaps, the most widely recognized unethical practice in medical writing is plagiarism. Plagiarism can be of different forms—some may be subtle and may not be classified under scientific misconduct. Others may amount to misappropriation and therefore to scientific misconduct that can have both ethical and legal implications.

### Definition

The use of the word “plagiarism” in the English language dates back to the 1600s. It is derived from the Latin word *“plagiare”* which means to “kidnap.”

The World Association of Medical Editors (WAME) defines plagiarism as[1]

“… the use of others’ published and unpublished ideas or words (or other intellectual property) without attribution or permission, and presenting them as new and original rather than derived from an existing source.”

### Why is “plagiarism” such a big issue?

Plagiarism may be detected at the level of the author, the reviewer, or the editor. A first-hand proofreading by the author himself sometimes alerts him of any inadvertent plagiarism. In such cases, the author simply corrects his mistake before submitting the manuscript to the journal. Sometimes, after the author has submitted the manuscript, he may realize that certain portions of text he had written may be deemed as plagiarized. He can then write an apology to the concerned editor and halt the publication.

The reviewer is a facilitator both for the editor and the author. He helps the editor by providing a critical, scientific, and a fair review of the manuscripts. The reviewer also helps the author by making specific recommendations for revision. If a reviewer detects that a particular portion of the text is not appropriately or accurately referenced, he suggests the author to make amendments. The reviewer can also recommend rejection of the article, clearly explaining the reason for such rejection, when he detects plagiarism.

Depending on the reviewer and editor’s judgment, subtle forms of plagiarism, if unintentional, sometimes may not be labeled as scientific misconduct or require a legal sanction. The reviewer may simply point out the error and instruct the author to amend it.

However, in its more severe forms, especially when it is intentional, plagiarism is considered a serious offence. If an author is found guilty of such an offence, his article may be retracted. The journal’s editorial board may issue a publication ban on him. The case of plagiarism may be reported to his employers and/or the professional bodies that the author may be a member of. Once this happens, it could lead to loss of funding for his research, loss of professional dignity, and even loss of employment.

The final decision of rejection or acceptance lies with the editor of the journal. Even if a manuscript has been passed by the reviewer, the editor can still reject it if he detects fraud or plagiarism.

What happens when whole or part of an article is copied from another source, and editors overlook the instance and the article goes into print? In such a case, if plagiarism is detected at a later stage, it leads to loss of reputation of not only the author(s) but also the reviewers, editors, and the journal as a whole. This could affect other journals under the same publication banner. The very essence and public respect for science and its truthfulness is lost in the process.

Such retraction is not unknown in medicine. A manuscript[2] was retracted from the April 2010 issue of *Anaesthesia and Analgesia* when it was found to have been plagiarized from five other published manuscripts, one of which was written by an Indian author.[3] The editor-in-chief of the concerned journal had to publish a Notice of Retraction[4] in the December 2010 issue of the journal, apologizing since the instances of plagiarism had not been detected in the review process. Strange as it may seem, the same issue also carries a Request for Retraction[5] by the concerned Indian author after a 2008 manuscript, where she was one of the lead authors,[6] was found to have been partly plagiarized from another 2004 manuscript[7] published in *Canadian Journal of Anesthesia*. Disciplinary action may be taken against the plagiarist. On being found guilty of plagiarism, a practicing psychiatrist and radio and television broadcaster in London had to step down as director of the Centre for Public Engagement in Mental Sciences in the institute where he was employed and was suspended from practice for three months by the General Medical Council.[8,9]

## TYPES OF PLAGIARISM

Plagiarism can be of several types. In this article, we present the more common varieties.

### Plagiarism of ideas

Even if an author does not copy any words and phrases from the original article, if he simply uses the same idea, thought, or invention and presents it as his own without proper acknowledgment, the same may amount to plagiarism. This kind of plagiarism is difficult to detect[10] but once detected, it is as serious an offence.

When can plagiarism of an idea occur? Here are a few examples that come to mind. It is possible that after a particular article has been rejected by a board of reviewers, one of the reviewers may “kidnap” the idea, write a fresh article, and get it published in a different journal under his name. This is plagiarism of idea.

Another kind of plagiarism is commonly seen among postgraduate students who borrow ideas for their thesis papers after searching through earlier research papers. Faculty members at some institutes quite often do not mind such practices and there have been instances where the student has been asked by his/her guide to pick up a thesis that is over 4 to 5 years old and present the study as a new one. Are they not encouraging plagiarism by doing so?

Plagiarism of ideas is also common during seminar and conference presentations. The presenters often pick up ideas from various sources such as text books, research journals, conference proceedings, etc., and compile a presentation on a particular topic and present it as their own. As long as all sources are aptly acknowledged in the presentation, it is fair play. However, any idea or thought that is not adequately attributed to its rightful owner, whether intentionally or unintentionally amounts to plagiarism of ideas.

SM Sapatnekar, in an editorial published in the *Journal of Association of Physicians of India*,[11] points out that a lot of plagiarism occurs in the medical field because of the “publish- or- perish mantra” adopted by researchers. Because research scholars and faculty members at an institute are expected to have a number of publications under their belt, many often reach out for the easier way out by plagiarizing ideas from previously published or unpublished works.

### Plagiarism of text (direct plagiarism)

Plagiarism of text is also called“word-for-word”plagiarism.[10] Roig[12] describes this kind of plagiarism as“…*copying a portion of text from another source without giving credit to its author and without enclosing the borrowed text in quotation marks.”*

Earlier, plagiarizing text from an article also required considerable hard work. One had to visit libraries and go through volumes of literature and read several textbooks to be able to copy relevant ideas and text. Even access to such resources was limited. Today, with the advancement of technology, plagiarism is easy. The practice seems to have increased manifold due to readily available internet access, simply because information is easily available online which can then be copied.“Cut-copy-paste ”seems to be happening across the world and is significantly prevalent in India as well. We have to understand that though technology makes plagiarism easy, it also makes detection of plagiarism even easier. There are both paid and free online software that can easily detect even short phrases that are copied verbatim from the original source. Sometimes, simply a Google search is sufficient to detect plagiarism.[13] Some other editors use active software such as SafeAssign^™^,[14] WCopyFind^™^,[15] and CrossCheck^™^.[16]

One does not need to copy entire paragraphs or even a single sentence to be guilty of plagiarism. Copying of a few phrases only can also amount to plagiarism of text. If a reviewer places two documents together and it is obvious that some text in one has been copied from the other, he can then label it as a plagiarized article. In Sapatnekar’s words,[11] “*To do this (plagiarism) overtly demonstrates your brashness. To do it covertly amounts to cowardice. To do it efficiently qualifies as an expertise; since ultimate success of a theft essentially lies in the theft passing undetected.*”

### Mosaic plagiarism

The American Medical Association Manual of Style[17] describes mosaic plagiarism as“… *borrowing the ideas and opinions from an original source and a few verbatim words or phrases without crediting the original author. In this case, the plagiarist intertwines his or her own ideas and opinions with those of the original author, creating a confused, plagiarized mass.*”

This is the more common form of plagiarism. The sentence or paragraph structure is almost similar to the original source with a few words and phrases here and there which are in the author’s own words. When the original author is not acknowledged and the reference not cited properly, such interlacing amounts to plagiarism. When the referencing is proper and quotation marks are adequately used, it is clear which of the words are by the author and which are from a different source. Unless this is done honestly, it could leave the reader confused.

### Self-plagiarism

If plagiarism is theft, self-plagiarism refers to stealing one’s own work. There is controversy on whether self-plagiarism actually amounts to scientific misconduct.[18]

Sometimes, the author may borrow significantly from his/her own previous work. In scientific journals, the reader expects to read original articles and any kind of self-plagiarism violates such expectations of the reader. However, suppose an author has been writing textbooks on, say Anatomy or Physiology, how does he make sure that no portion of what he has written before is repeated in his future books? He simply cannot change the human anatomy or physiology in order to avoid being labeled as a“self-plagiarist.” So what does he do? Common consensus is that he should seek permission for reproduction from the copyright holder (if the copyright has been transferred to the publisher). He must cite the original book, chapter, or article properly. The key here is disclosure and transparency in doing so. As long as he lets the editors and the end readers know that the work had been published elsewhere and provides proper citation for them to be able to judge which portions have been reused in the new article or chapter, he is being transparent and this could be considered fair play.

A member of WAME’s Ethics Committee says:[19] “*With respect to the issue of how much overlap is too much…a rule of thumb that some editors have applied when considering the amount of overlap between two review articles (not book chapters) has been overlap of more than one-third of the material.*”

The“publish-or-perish mantra ”adopted by academics and research scholars may be a step toward better prospects for a promotion or reward. But, it results in huge volumes of duplicate or redundant publications since each researcher tries to publish as many papers as he possibly can.

Some authors may submit the same article for publication in different journals without clearly mentioning to each group of editors that they are doing so. They do so to increase their chances of publication. This is unethical. Even a substantial overlap with a previously published article, without acknowledging the publication and the original authors, can amount to plagiarism and copyright infringement.

Duplicate or redundant publication may be seen in several forms.[8,12,20,21] The authors may be the same or the order of authorship may change. The same study sample, control data, or study outcomes may or may not be presented. Sometimes, writers use the same tables or figures that may have appeared in previous publications. Another common form of self-plagiarism is *salami slicing*. Instead of publishing a large study as a single article, sometimes authors “slice” it into several smaller articles. Should very large, complex longitudinal studies with multiple measures designed to test multiple related hypotheses be then published as a single article? What does one do when there is tight restriction on the length of a single manuscript? The editor can take a call on publishing very long manuscripts and short reports as needed. To improve the reporting of clinical trials, many journals now follow the CONSORT (CONsolidated Standards of Reporting Trials) 2010 checklist.[22]

Sometimes, authors submit the same article to two or more different journals within a few weeks’ gap. In case both the journals have published it, the article that was published later has to be retracted.[23,24] National Library of Medicine reserves the right to assign both the articles the Publication Type of Duplicate Publication [PT]. It can do so with or without prior notification to the authors or the editors. A PubMed search for“Duplication Publication [PT]” in February 2011, threw up at least 957 results.

If an article has originally been published in a non-English journal and the author wishes to publish a translation in an English journal for wider circulation or he wants to republish an article from an English journal in a local language, he needs to follow the Uniform Requirements issued by the ICMJE (International Committee of Medical Journal Editors).[25] He must mention his intentions clearly to the editors and the editors of both journals must agree. As his responsibilities toward the reader, the medical writer should cite the original article aptly.

The problem with duplicate publication is not just the ethical issues involved. There is wastage of resources. For each article that is accepted for publication, editors have to reject or delay the publication of several other articles due to lack of space. Duplicate publication of studies can lead to erroneous meta-analysis and distort the evidence base of clinical decision making. This can have a large impact on the healthcare delivery.

Plagiarism is extensively prevalent in the academic community. In an editorial, Satyanarayana[26] says,“*plagiarism begins very early in science*.” It probably starts with the seminar presentations students make early during their professional studies. Most theses/dissertations submitted by medical students are copied from previously published material. A Croatian study[15] showed that over 90% of medical students in their second year plagiarized to some extent when asked to write essays on given topics. Another study in a Boston university[27] found that one in 20 medical residency applications contained plagiarism. A look at some of the cases listed at the website of the Society for Scientific Values[28] shows that plagiarism is just as common in India as in any other country.

## COMMON TIPS FOR AVOIDING PLAGIARISM

- Ethical medical writers must always acknowledge the original source of the idea, text, or illustration.
- They must remember to enclose within quotation marks, all the text that has been copied verbatim from another source.
- When paraphrasing, they must read the text, understand completely, and then use only their own words.
- Even when explaining somebody else’s ideas in their own words, it is important that they properly acknowledge the original source.
- When not sure if the idea/fact they wish to include is common knowledge, a medical writer must cite references.
- They must cite references accurately. The writer must read the instructions to authors to know what style they need to use. Biomedical journals commonly use the Vancouver style.[29] Some textbook publishers prefer the Harvard referencing[29] style. Insufficient and inaccurate acknowledgement can also amount to plagiarism.
- A medical writer should avoid writing multiple separate articles if he can present a large complex study in a cohesive manner in a single article.
- Along with the manuscript, he should submit a cover letter to the editor, clearly stating any instances of overlapping from previous publications and asking for advice.
- Last, but not the least, if he feels he has unintentionally used somebody else’s ideas or text without appropriate referencing, he needs to write to the editor of the journal for advice. Confession is always better than to be caught stealing.

## References

[1] World Association of Medical Editors. Publication ethics policies for medical journals.

[2] Memis D, Inal MT, Temizoz O, Genchallac H, Ozdemir H, Sut N (2010). The effect of celiac plexus block in critically ill patients intolerant of enteral nutrition: A randomized, placebo-controlled study. Anesth Analg.

[3] Bhatnagar S, Gupta D, Mishra S, Thulkar S, Chauhan H (2008). Bedside ultrasound-guided celiac plexus neurolysis with bilateral paramedian needle entry technique can be an effective pain control technique in advanced upper abdominal cancer pain. J Palliat Med.

[4] Shafer SL (2010). Notice of retraction. Anesth Analg.

[5] Bhatnagar S (2010). Request for retraction. Anesth Analg.

[6] Gupta D, Jain R, Mishra S, Kumar S, Thulkar S, Bhatnagar S (2008). Ultrasonography reinvents the originally described technique for ganglion impar neurolysis in perianal cancer pain. Anesth Analg.

[7] Munir MA, Zhang J, Ahmad M (2004). A modified needle-inside-needle technique for the ganglion impar block. Can J Anaesth.

[8] BBC News. Media doctor admits to plagiarism.

[9] The Guardian The Guardian. Persaud suspended from practice for three months over plagiarism.

[10] Singh AJ (2007). Plagiarising plagiarism. Indian J Community Med.

[11] Sapatnekar SM (2004). Plagiarism. J Assoc Physicians India.

[12] Roig M Avoiding plagiarism, self-plagiarism, and other questionable writing practices: A guide to ethical writing. http://facpub.stjohns.edu/~roigm/plagiarism/plagiarism%20of%20text.html.

[13] Weeks AD (2006). Detecting plagiarism: Google could be the way forward. BMJ.

[14] Chaudhuri J “Deterring digital plagiarism, how effective is the digital detection process?” Webology,5(1), Article 50. http://www.webology.ir/2008/v5n1/a50.html.

[15] Bilić-Zulle L, Frković V, Turk T, Azman J, Petrovecki M (2005). Prevalence of plagiarism among medical students. Croat Med J.

[16] Zhang H (2010). Cross Check: An effective tool for detecting plagiarism. Learn Publ. Availablefrom:http://www.zju.edu.cn/jzus/download/CrossCheck.pdf.

[17] Iverson C, Flanagin A, Fontanarosa PB, Glass RM, Glitman P, Lantz JC (1998). American Medical Association Manual of Style. A Guide for Authors and Editors.

[18] Broome ME (2004). Self-plagiarism: Oxymoron, fair use, or scientific misconduct?. Nurs Outlook.

[19] World Association of Medical Editors. Self-plagiarism of textbook chapters. [Ethics Resource].

[20] Cicutto L (2008). Plagiarism: Avoiding the peril in scientific writing. Chest.

[21] Johnson C (2006). Repetitive, duplicate, and redundant publications: A review for authors and readers. J Manipulative Physiol Ther.

[22] The CONSORT Statement. http://www.consortstatement.org/consort-statement/overview0/.

[23] Yoda K (2010). Announcement from the Editor-in-Chief regarding duplicate publication. Retraction. Biosci Biotechnol Biochem.

[24] Shimokawa H (2010). Urgent announcement from the editor-in-chief regarding duplicate publication. Circ J.

[25] ICMJE Uniform Requirements for Manuscripts Submitted to Biomedical Journals:Publishing and Editorial Issues Related to Publication in Biomedical Journals:Overlapping Publications. http://www.icmje.org/publishing_4overlap.html.

[26] Satyanarayana K (2010). Plagiarism: A scourge afflicting the Indian science. Indian J Med Res.

[27] Segal S, Gelfand BJ, Hurwitz S, Berkowitz L, Ashley SW, Nadel ES (2010). Plagiarism in residency application essays. Ann Intern Med.

[28] Society for Scientific Values. Cases of misconduct investigated by SSV. http://www.scientificvalues.org/cases.html.

[29] British Medical Association. Reference styles: Harvard and Vancouver. http://www.bma.org.uk/library_medline/electronic_resources/factsheets/LIBReferenceStyles.jsp.

